# Tomasz Drobnik (1858–1901)

**DOI:** 10.1007/s00415-015-7816-2

**Published:** 2015-06-17

**Authors:** Andrzej Grzybowski, Krzysztof Pietrzak

**Affiliations:** Department of Ophthalmology, Poznan City Hospital, Poznan, Poland; Chair of Ophthalmology, University of Warmia and Mazury, Olsztyn, Poland; Department of Orthopaedics and Traumatology, University of Medical Sciences, 28 Czerwca 1956 135/147, 61-545 Poznan, Poland

Tomasz Drobnik was born on 6 September 1858 in Pleszew as a Polish citizen. He moved to Poznań where he completed his secondary education. In 1880 he started his medical studies in Wroclaw, Poland, and completed them in Würzburg, Germany. It was there where he obtained a degree as a doctor of medicine in 1885 [1]. After completing his military service, he worked in Wrocław and subsequently in Strasburg. The focus of his interest was thyroid surgery [2], which he continued between 1887 and 1890 guided by Professor J. Mikulicz-Radecki in the Surgical Clinic in Konigsberg.

Drobnik achieved supremacy in operational treatment of effects of neurological disorders. He proved the connection between the branches of vagus nerve and the sympathetic trunk [3]. He also described in detail the anatomy of the branch of the sympathetic trunk [3].

Drobnik was one of the world pioneers of the facial nerve surgery. He made a successful connection of the facial nerve with the accessory nerve. He made it after its paresis caused by the abscess of the temporal bone. After several months he observed that the facial features became more symmetrical. Nevertheless, the exact date of the operation still stirs controversies. In all probability it was performed between 1887 and 1890. Drobnik worked in Konigsberg at that time and focused on theoretical aspects of the facial nerve. He also described an operation of harelip which was later described in his publication [4]. Mikulicz allowed him to conduct the operation only once, similarly to the operation of the facial nerve. A suggested date of 1879 is impossible to accept as Drobnik was only 21 at that time and was not a medical student yet. In 1894 an unsuccessful attempt to reconstruct a facial nerve was undertaken by Charles Ballance, whose intention was to shorten its course. Difficulties in establishing the date of Drobnik’s operation do not allow him to be credited with superiority in performing a facial nerve surgery. Undoubtedly, however, he should be considered the first one to have conducted the surgery successfully.

Drobnik focused on treatment and theoretical examination of effects of neurological disorders. He was the first to notice that the club foot is the result of muscle imbalance, particularly the imbalance of the rear shin splint and the musculus flexor digitorum longus [5]. He also noticed that clawed fingers are the result of disorders in musculus flexor digitorum longus. Drobnik also established the method of treatment of the clubbed foot resulting from the peroneal nerve palsy. Such approach to disorders presently termed as neuro-orthopedic disorders was absolutely innovative at that time.

Drobnik was the first to perform successfully the transplantation of tendons. It was as groundbreaking as the first tendon cut by Louis Stromeyer (1804–1876) in 1831. The operation was performed on a 7-year-old girl treated for effects of poliomyelitis. Drobnik transplanted the extensor hallucis longus onto extensor hallucis brevis with a good clinical result. He presented the result on 4 December 1892 at a meeting of the Medical Department of the Poznań Society of Friends of Sciences. An attempt at tendon transplantation had been undertaken a year earlier by Carl Nicoladoni (1847–1902); it was however unsuccessful. Drobnik subsequently presented descriptions of operational treatment of paralytic foot drop as an effect of infantile cerebral palsy [6, 7]. In 1894 Drobnik was the first to transplant a tendon by implanting it directly into the bone. He also performed the first transplantation of tendons within the upper limb on a 14-year-old girl after radial nerve palsy. The girl regained the ability to grasp objects. The operation was performed despite the then common belief that radial nerve palsy is resistant to treatment. In the course of rehabilitation Drobnik paid attention to such details as the regaining of proprioception by touching the floor with a bare foot [8].

In 1890 Drobnik returned to Poznań where he took the position of the Head of Department of Paediatric Surgery. He was in charge of a 12-bed ward in extremely difficult conditions, initially without any assistant. He made a series of surgical discoveries there. He worked out a method of treating cleft palate, pioneered the treatment of diphtheria by cutting the windpipe and found a new way for treating large abdominal ruptures. He put an emphasis on the importance of fluid intake in surgical treatment, and on the operational treatment of peritonitis. The last two observations were in conflict with commonly accepted doctrines at the time [4, 9, 10]. Drobnik can be credited with a series of orthopedic discoveries. He forged the way to multiple-stage treatment of the club foot, lower limb dysfunctions, and in bone and joint tuberculosis. He was an affirmed opponent of applying tuberculin in the treatment of tuberculosis. His new techniques of wound treatment gained wider recognition only years later, during the First World War.

In 1899 he took the Chair of the Surgery Department in Poznań City Hospital, which was decided upon by the then German authorities. They appreciated Drobnik’s undisputable achievements in spite of their utterly anti-Polish policy. Drobnik was deeply involved in voluntary work and worked in a number of positions. He died of a heart attack on 22 May 1901. His funeral was attended by hundreds of people of many nationalities. Drobnik was married to Helena nee Szuman. They had three sons.
